# Birth dates vary with fixed and dynamic maternal features, offspring sex, and extreme climatic events in a high‐latitude marine mammal

**DOI:** 10.1002/ece3.1985

**Published:** 2016-02-24

**Authors:** Jay J. Rotella, J. Terrill Paterson, Robert A. Garrott

**Affiliations:** ^1^ Ecology DepartmentMontana State UniversityBozemanMontana59717

**Keywords:** Antarctica, birth dates, birth timing, maternal effects, population ecology, Weddell seals

## Abstract

Reproductive synchrony tends to be widespread in diverse species of plants and animals, especially at higher latitudes. However, for long‐lived mammals, birth dates for different individuals can vary by weeks within a population. A mother's birth timing can reveal useful information about her reproductive abilities and have important implications for the characteristics and survival of her offspring. Despite this, our current knowledge of factors associated with variation in birth dates is modest. We used long‐term data for known‐age Weddell seals in Antarctica and a Bayesian hierarchical modeling approach to study how birth dates varied with fixed and temporally varying features of mothers, whether sex allocation varied with birth timing, and annual variation in birth dates. Based on birth dates for 4465 pups born to 1117 mothers aged 4–31, we found that diverse features of mothers were associated with variation in birth dates. Maternal identity was the most important among these. Unlike most studies, which have reported that birth dates occur earlier as mothers age, we found that birth dates progressively occurred earlier in the year in the early part of a mother's reproductive life, reached a minimum at age 16, and then occurred later at later ages. Birth dates were positively related to a mother's age at primiparity and recent reproductive effort. The earliest birth dates were for pups born to prime‐age mothers who did not reproduce in the previous year but began reproduction early in life, suggesting that females in the best condition gave birth earlier than others. If so, our finding that male pups tended to be born earlier than females provides support for the Trivers–Willard sex‐allocation model. Average birth dates were quite consistent across years, except for 2 years that had notable delays and occurred during the period when massive icebergs were present and disrupted the ecosystem.

## Introduction

In diverse species of plants and animals, reproduction is clustered temporally, especially at higher latitudes where seasonal changes in climate as well as biotic interactions are considered important drivers of synchrony (Ims 1990). Timing of reproduction can have important consequences for survival of offspring and their parents because of temporal variation in food availability, ambient temperatures and resulting energy budgets (Festa‐Bianchet 1988a; Bronson 2009). Accordingly, numerous studies have investigated the factors related to individual‐ and population‐level variation in timing of reproduction (McGinnes and Downing 1977; Guinness et al. 1978a; Plard et al. 2014).

For long‐lived mammals in temperate zones, populations with strongly synchronized reproduction can have important levels of individual variation in birth dates (Plard et al. 2013), which can in turn allow for population‐level plasticity in birth timing under different annual conditions (Cordes and Thompson 2013). The date at which a female gives birth can vary with her age, reproductive experience, and body condition (Festa‐Bianchet 1988b; Sydeman et al. 1991; Lunn and Boyd 1993; Plard et al. 2014). Even after such factors are considered, some individuals repeatedly give birth earlier or later than others (Cordes and Thompson 2013; Plard et al. 2013; Wolcott et al. 2015). Thus, birth timing for a given population depends on the individuals in the population and the environmental conditions those individuals face (Sydeman et al. 1991; Boyd 1996; Cordes and Thompson 2013; Friebe et al. 2014).

As reviewed by Wolcott et al. (2015), birth timing is hypothesized to depend on maternal condition, which in turn depends on an individual's ability to efficiently acquire nutritional resources. Resource acquisition is often found to improve early in life, peak for prime‐age individuals, and possibly decline thereafter (Gaillard et al. 2000). Given this, it is not surprising that the number of studies that have reported a negative relationship between maternal age and birth date (Reiter et al. 1981; Lunn and Boyd 1993; Boltnev and York 2001; Haskell et al. 2008; Wolcott et al. 2015) greatly outnumbers those that have not found a relationship (Guinness et al. 1978a; Ellis et al. 2000). Rarer still are studies that have reported a curvilinear relationship where a mother gives birth at later dates early and late in her reproductive life and at earlier dates at intermediate maternal ages (Sydeman et al. 1991). It is difficult to know whether senescent changes in birth dates are rarely reported because the phenomena are actually uncommon or because most studies do not contain large enough samples of data for old‐age individuals to detect changes that occur late in life (Clutton‐Brock and Sheldon 2010).

At the population level, age alone might not be an adequate predictor of birth date for species with flexible reproductive schedules because (1) females of the same age can have different levels of reproductive experience (Sydeman et al. 1991) and (2) those that have foregone reproduction for a year or more might have had more opportunity to store body reserves. Hence, reproductive state in the previous year can be an important predictor of birth date. For example, birth dates have been found to be later for mothers that reared young in the previous year compared to dates for those that did not (Guinness et al. 1978b) and for primiparous versus multiparous mothers (Maniscalco et al. 2006). Environmental conditions faced by mothers just prior to embryonic implantation or during gestation can also affect their body condition and birth timing (Boyd 1991; Pitcher et al. 2001). For example, studies of diverse species of large mammals have reported delays in parturition in years with low food abundance (McGinnes and Downing 1977; Lunn and Boyd 1993; Boyd 1996; Soto et al. 2004; Haskell et al. 2008; Wolcott et al. 2015).

Despite evidence that birth dates change predictably as dynamic female traits (e.g., age and reproductive experience) change over a lifetime, birth dates for individual mothers have also been reported to be repeatable in large mammals in studies conducted in the wild (Guinness et al. 1978b; Ellis et al. 2000; Cordes and Thompson 2013; Plard et al. 2013) and in captivity (Temte 1991; Wolcott et al. 2015). Such repeatability could be tied to individual variation in acquisition of nutritional resources. However, the causal mechanism must be somewhat complex given that a recent study in which captive females were experimentally kept at a high nutritional plane found that maternal identity accounted for more variation in birth timing than did other biological or environmental covariates (Wolcott et al. 2015). Despite uncertainty about the underlying causes of individual repeatability in parturition date, it is clear that studies of birth timing should consider both dynamic maternal features and maternal identity.

Just as the characteristics of mothers have been shown to vary by birth date, so too have the attributes of young. For example, body mass at parturition, weight gain during lactation, and subsequent survival in the early years of life have received much attention and been related to birth dates in many informative studies (Guinness et al. 1978b; Festa‐Bianchet 1988a; Boltnev and York 2001; Côté and Festa‐Bianchet 2001; Feder et al. 2008; Marcil‐Ferland et al. 2013; Plard et al. 2015). Sex ratios of offspring born on different dates have also proven to be a particularly interesting subject of study. For long‐lived, polygynous mammals, reproductive success is more variable for males than females, and females of superior quality are predicted to produce more male offspring than females of lower quality (Trivers and Willard 1973). Among the many studies of variation in mammalian sex ratios, those that measured maternal condition near conception consistently indicate that mothers in better condition are more likely to produce sons (Cameron 2004). Further, females in better condition have been reported to give birth earlier in a number of species (Reiter et al. 1981; Lunn and Boyd 1993; Boltnev and York 2001; Robbins et al. 2012; Plard et al. 2014), and several studies have found a male‐biased sex ratio in earlier‐born offspring (Coulson and Hickling 1961; Stirling 1971; Hemmer 2006; Holand et al. 2006). However, many other studies have failed to find evidence of relationships between birth date and offspring sex ratios (Gaillard et al. 1993; Boyd 1996; Boltnev and York 2001; Haskell et al. 2008). Additional studies of how birth timing varies with offspring sex and maternal characteristics in long‐lived polygynous species are needed to better determine how broad the empirical support is for the Trivers and Willard (1973) sex‐allocation model.

**Figure 1:**
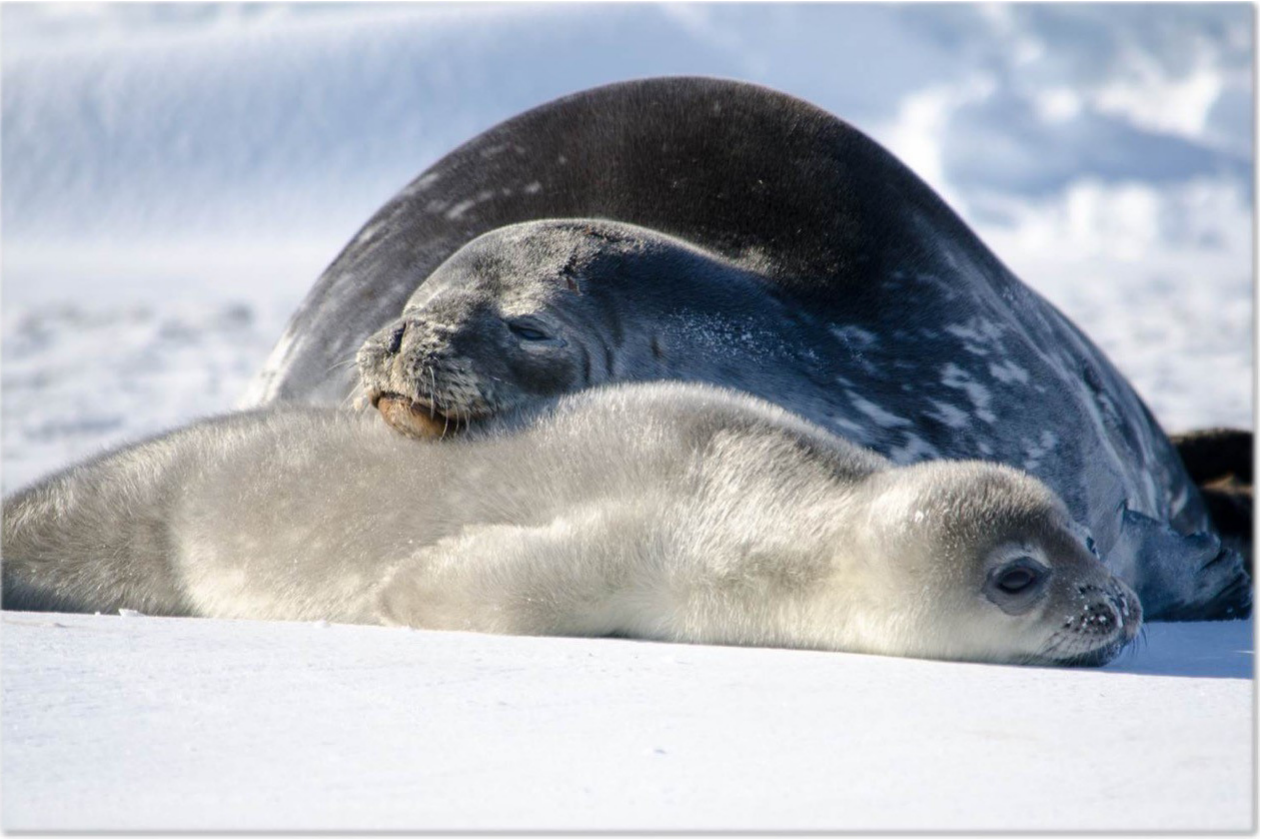
Female Weddell seal (*Leptonychotes weddellii*) with her pup, in Erebus Bay, McMurdo Sound, Antarctica. Photograph credit: W.A. Link. Image was obtained under NMFS Permit No. 1032‐1917.

Here, we evaluate diverse sources of variation in birth dates of Weddell seals using data for 4465 pups and 1117 known‐age mothers collected over 28 years. Weddell seals are long‐lived predators of the Southern Ocean that are ideal for studying variation in birth dates (Fig. 1). Selection on birth date is expected to be especially strong in this southern‐most population of mammal. Pups are highly visible and approachable in aggregations at traditional birthing colonies. Accordingly, birth dates can be obtained precisely for most pups unless weather or other logistical constraints prevent access to colonies within a few days of birth. Pups can be reliably associated with their mothers during the 5‐ to 6‐week lactation period when mothers rely heavily on stored subcutaneous fat for milk production (Stirling 1969; Boness and Bowen 1996; Wheatley et al. 2006). In this work, the study population has been the subject of a long‐term mark–recapture study such that the ages and reproductive histories are known for most females in this highly philopatric population (Siniff et al. 1977; Hadley et al. 2007). We use the data to evaluate predictions about how birth date would vary with maternal characteristics, pup sex, and year. Specifically, we assessed whether pup birth dates (1) differed for male and female pups, (2) changed with dynamic maternal traits (age and reproductive state in the previous year), (3) varied with fixed maternal traits (age at first reproduction and a mother's identity), and (4) varied among years. Our predictions for the relationships and our justification for them are presented in the data analysis section where details are provided for each birth date covariate we evaluated.

## Material and Methods

### Study area and population

Data were collected along the west side of Ross Island in McMurdo Sound, Antarctica (−77.62° to −77.87°S, 166.3° to 167.0°E), in the 8–14 pupping colonies that form along perennial cracks in the sea ice caused by tidal movements and glacial pressure (see Cameron and Siniff 2004; for additional details). Each year, 300–600 pups are born on the sea ice during austral spring. Currently, approximately 80% of the seals in this population are marked, and over 80% of marked animals are of known age.

### Data collection

From 1969 until present, Weddell seals in the study area have been surveyed and individually marked with tags attached to the interdigital webbing of each rear flipper as part of a long‐term mark–recapture study (Siniff et al. 1977). From 1982 to present, all pups born in the study area have been tagged annually. From 1993 to present, each colony has been visited every 2–3 days throughout the birthing period, which allowed precise birth dates (as well as pup sex and mother's identity) to be determined for many pups per year. Prior to 1993, birth dates were only recorded when less frequent visits happened to coincide closely with a pup's day of birth. Also, in more recent years, inclement weather or logistical constraints have sometimes forced less frequent visits to occur. For the analyses presented here, we only used data from pups (1) whose birth dates could be precisely determined based on appearance of the pup, placenta, and/or umbilical stump and (2) whose mothers were of known age.

### Data analyses

We used a Bayesian‐based linear modeling approach to investigate relationships between a pup's birth date and (1) a pup's sex, (2) fixed and dynamic attributes of its mother, and (3) year. We include a binary covariate (PupMale) that indicated if a pup was male or not (prediction: negative in accordance with Stirling (1971)), who reported that male Weddell seal pups tend to be born earlier). Maternal attributes included a mother's age, age at first reproduction, reproductive status in the previous year, and identity. For maternal age, we used a quadratic functional form (i.e., *β*_age.l_MomAge + *β*_age.q_MomAge^2^) as it could accommodate (1) no change in birth date with increasing maternal age (if *β*_age.l_ = 0 and *β*_age.q_ = 0), (2) linear changes whereby birth dates progressively occur earlier (as is most commonly reported in the literature) or later with age (if *β*_age.q_ = 0), or (3) allow birth date to initially occur earlier with increasing maternal age until reaching a minimum at some prime age and to then occur later at older ages (if *β*_age.l_ > 0 and *β*_age.q_ < 0, as we predicted given the wide range of ages our data encompassed). Age at first reproduction (AgeFirst), which varies in Weddell seals and is predicted to be inversely related to female quality (Hadley et al. 2006), was modeled using a linear functional form (prediction: negative slope). A female's reproductive status in one season may affect her ability to invest in future reproduction either negatively through reproductive costs (Guinness et al. 1978a; Festa‐Bianchet 1988a; Hadley et al. 2007) or positively if females that reproduce often are also those that are better at acquiring resources for recovering from past reproduction and preparing for future reproduction (Clutton‐Brock 1984). We therefore included a categorical measure of a female's reproductive status in the previous year using the categories prebreeder (Pre), first‐time breeder (FirstTime), an experienced breeder (Exper), or a skip breeder (Skip) (prediction: earlier birth dates for mothers who were Pre or Skip in previous year; latest dates for those that were primiparous in previous year). Both female parity and maternal age have been used in models for the timing of parturition in northern elephant seals (*Mirounga angustirostris*), with parity reported to be more strongly associated than age to birth date in that species (Sydeman et al. 1991). In our study, the high correlation between parity and maternal age (0.88) renders the simultaneous evaluation of those covariates difficult. Thus, we chose to use maternal age to specifically assess the strength of evidence for age‐related changes in parturition date, with the understanding that maternal age is a reasonable proxy for parity. To evaluate repeatability of birth dates for individual mothers, we included a random effect of maternal identity (normally distributed intercept adjustments with mean = 0; prediction: variance >0 but no prediction on its magnitude given lack of previous information). Finally, to evaluate annual variation in average birth dates among years, we included a random effect of year (normally distributed intercept adjustments with mean = 0; prediction: variance >0 but modest given our observations of how repeatable peak of birthing has been).

We ran a single model that included the modest set of covariates listed above and which assumed errors in birth dates were normally distributed about the mean. Birth dates, *B*_*i,j,k*_ (observation on pup *i*, with maternal identity *j*, and in year *k*), were treated as independent normal random variables with the mean *μ*_*i,j.k*_ a function of the explanatory variables:

$$\begin{array}{cc} \mu_{i,j.k}=\alpha & +\beta_{\mathrm{sex}}\times {\text{PupMale}}_{i}+\beta_{\mathrm{age}.l}\times {\text{MomAge}}_{i}+\beta_{\mathrm{age}.q}\times {\text{MomAge}}_{i}^{2}+\beta_{\mathrm{agefirst}}\times {\text{AgeFirst}}_{i}\\ & +\beta_{\mathrm{Pre}}\times {\text{Pre}}_{i}+\beta_{\mathrm{FirstTime}}\times {\text{FirstTime}}_{i}+\beta_{\mathrm{Exper}}\times {\text{Exper}}_{i}+\beta_{\mathrm{Skip}}\times {\text{Skip}}_{i}+\eta_{j}+\gamma_{k} \end{array},$$

where *η*_*j*_ is the random effect of maternal identity, and *γ*_*k*_ is the random effect of year. We used a single model because of the large amount of literature regarding factors related to variation in birth rates in long‐lived mammals; our ability to include a variety of features about mothers, pups, and years, which included random effects that allowed for individual and annual variation; and comments by Ver Hoef and Boveng (2015). Rather than conduct model selection to attempt to pare the model down, we instead estimated the coefficients associated with each of the covariates to assess the support in the data for a strong, weak, or no relationship between birth date and each of the covariates in question. To do so, we first considered the point estimate and credible interval for each of the parameters in the model. We also considered model‐based predictions and their associated uncertainties across the observed range of covariate values. Stronger relationships were evidenced by point estimates that were further from zero, credible intervals that were narrow and did not overlap zero, and predicted birth dates that varied by at least several days across the range of observed values for the covariate in question. Although we do not know exactly what effect size to consider biologically significant, a difference of a few days could be important in this species given the rapid rate at which pups gain mass per day (Wheatley et al. 2006) and the rapid deterioration of sea ice in the study area late in the lactation period. Pups born a few days later than others would be expected to be 5–8 kg lighter on a given date late in lactation and thus might also be less likely to survive the early independence periods when marine predators gain access to pupping colonies during ice breakup. The continuous covariates (MomAge and AgeFirst) were centered using their mean value and scaled by two standard deviations to make them more directly comparable to binary covariates (Gelman 2008). MomAge^2^ was constructed from the scaled values of MomAge.

Bayesian hierarchical models require distributional assumptions for coefficients in each level of the model. To penalize for any collinearity between the covariates and assist in the interpretation of the results, we investigated the use of several shrinkage priors in a Bayesian interpretation of regularization for the regression coefficients for the fixed effects (Hooten and Hobbs 2014). The specific priors evaluated were the Bayesian lasso (Park and Casella 2008), ridge (Hoerl and Kennard 1970), and horseshoe (Carvalho et al. 2009). The results obtained using various shrinkage priors and those obtained using diffuse, uniform priors were very similar. We therefore used independent, improper uniform priors for the intercept and each of regression coefficients for the covariates. The random effects of maternal identity (*η*) and year (*γ*) in the model were treated as independent, normal random variables with means equal to zero, and variances *σ*_*η*,_ *σ*_*γ*_. The variances for the random effects were assigned diffuse uniform priors on the interval (0,100).

We fit all models using the rstan package in the R programming language (R Development Core Team 2015), a package that utilizes the STAN modeling language to implement MCMC sampling (Stan Development Team 2014). Four chains were run for each model with 10,000 samples per chain used for posterior inference after discarding 5000 samples as burn‐in. Posterior convergence was assessed both graphically and with the Gelman–Rubin statistic, $\hat{R}$ (convergence assumed for values $\hat{R}$ < 1.01). We assessed model fit using a hierarchical implementation of the classical *R* ^2^ (Nakagawa and Schielzeth 2013) and by assessing plots of residuals against fitted values. We also examined plots of residuals to look for evidence of goodness‐of‐fit problems. For each of the estimated coefficients, we developed 95% highest density intervals (HDI) based on the posterior sample (Hyndman 1996).

## Results

Birth dates were available for 4465 pups produced by 1117 different mothers. The mean number of birth dates available per mother was 4 (SD = 2.9, range = 1–18). On average, mothers were aged 12 years (SD = 4.29, range = 4–31, *n*_age ≤ 10_ = 1858, *n*_age ≥ 20_ = 321). Average age of first reproduction was 7.5 years (SD = 1.5, range = 4–16). The data included birth dates for 902, 406, 2036, and 1121 mothers whose reproductive state in the previous year was Pre, FirstTime, Exp, or Skip, respectively. The overall proportion of pups that were male (*n* = 2197) was 0.49 (95% CI = 0.48–0.51; female proportion = 0.51, 95% CI = 0.49–0.52, *n* = 2268).

Birth dates from 28 different years were used in the analyses (mean number per year = 159.5, SD = 102.7). Although samples were modest prior to 1993 (mean number per year = 18.6, range = 2–45), data from those years were included to maximize the number of birth dates recorded per mother. Data from 2013 were not included in the analysis because logistical constraints prevented us from collecting birth dates during the first half of the birthing season. From 1993 through 2014, the average birth date was 30 October (SD = 7.1), and, on average, the annual birthing season was 36.5 days long (SD = 4.4), began on 14 October (SD = 2.9), and ended on 19 November (SD = 3.9). Based on the lower and upper quartiles for birth dates in each year, half of the births occurred in an average span of 9.5 days each year (SD = 2.9) from 24 October (SD = 2.9) through 2 November (SD = 2.9).

**Figure 2:**
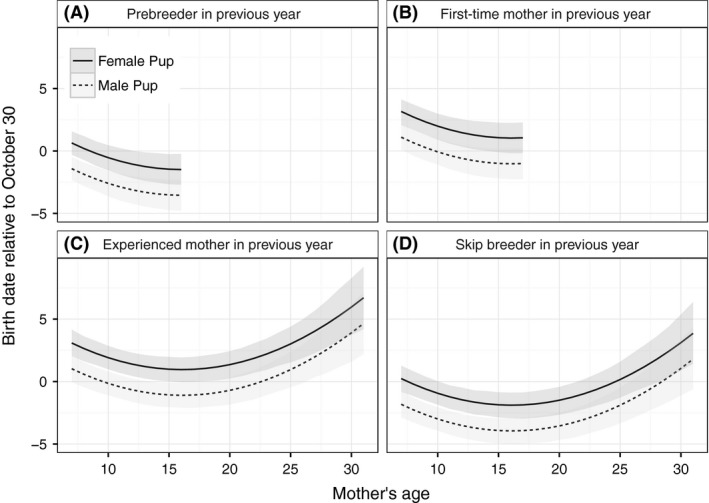
Estimates of birth dates for female (solid line) and male (dashed line) Weddell seal pups born to mothers of different ages based on Bayesian hierarchical modeling of data from 4465 pups born from 1984 through 2014 in Erebus Bay, McMurdo Sound, Antarctica. Predicted values are based on mothers that first reproduced a pup when they were aged 7 years, assuming an average maternal effect and year effect of zero. Panels present birth date predictions for mothers that were in different reproductive states in the previous year: (A) Mothers that were prebreeders in the previous year are those producing their first pup in the current year; (B) mothers that were first‐time mothers in the previous year are those producing their second pup in the current year, which immediately follows their year of primiparity; (C) mothers that were experienced mothers last year are those giving birth to at least their third pup in the current year and having pups in back‐to‐back years; and (D) mothers that skipped reproduction last year are those that previously produced a pup but not in the previous year. Predictions are presented only for those ages that occur in the dataset for mothers in a given panel's reproductive category. Gray bands present uncertainty about predictions based on the posterior distribution and display the most credible set of values that contain 95% of the posterior distribution's mass.

Estimated parameters for the model of variation in birth dates were in agreement with most but not all of our predictions and provided several novel insights. In the deterministic portion of the model, results supported our predictions that birth dates would be earlier for sons than daughters and earlier for pups born to mothers in their late teens than for younger or older mothers (Table 1, Fig. 2). Male pups were born 2.1 (95% HDI: −2.4, −1.8) days earlier than female pups. When each birthing season was broken into four 12‐day intervals that encompassed all but one of the birth dates across all years, the proportion males was 0.68 (SE = 0.02) from 11 to 22 October, 0.49 (SE = 0.01) from 23 October to 3 November, 0.40 (SE = 0.01) from 4 to 15 November, and 0.36 (SE = 0.06) after 15 November. Across the range of maternal age, birth dates varied by approximately 5 days after controlling for other covariates, for example, experienced mothers aged 16, 9, and 30 years old that had an age of primiparity of 7 years are predicted to have birth dates of 30 October (95% HDI: 29 October to 31 October), 31 October (30 October to 1 November), and 4 November (2 November to 6 November) for daughters.

**Table 1:** Parameter estimates and associated standard errors and quantiles for a model explaining variation in birth date for Weddell seal pups (*n* = 4465) in McMurdo Sound, Antarctica, based on characteristics of the pup's mother (her age and age squared, age at first reproduction, and reproductive status in the previous year [Pre, First, Exper, or Skip]), the pup's sex (was it a male), and the year. In the model, continuous covariates were standardized (mean = 0, SD = 2) so that coefficients associated with continuous covariates were more directly comparable to binary covariates (Gelman 2008). The model's intercept provides an estimate of the average birth date for the reference group (mother was in the Pre state in the previous year and produced a female pup in the current year) for a mother with average values for all continuous covariates, that is, her standardized covariate values were 0. Random effects represent estimates of the process standard deviation associated with maternal identity (*σ*_Mother_) and year (*σ*_Year_). For each variable in the model, we present the estimated coefficient, SE, and 95% highest density interval (HDI) based on the mean, standard deviation, and distribution of values in the posterior distribution

Variable	Estimate	SE	95% HDI
Intercept	−0.88	0.55	−1.97, 0.14
MomAge	−1.78	0.39	−2.53, −1.02
MomAge^2^	1.90	0.33	1.22, 2.53
AgeFirst	1.14	0.34	0.46, 1.80
PupMale	−2.06	0.15	−2.36, −1.77
First	2.52	0.31	1.89, 3.11
Exper	2.44	0.34	1.80, 3.15
Skip	−0.41	0.33	−1.04, 0.24
*σ*_Mother_	5.02	0.14	4.75, 5.30
*σ*_Year_	1.94	0.32	1.37, 2.57
*σ*_*y*_	4.64	0.06	4.53, 4.75

**Figure 3:**
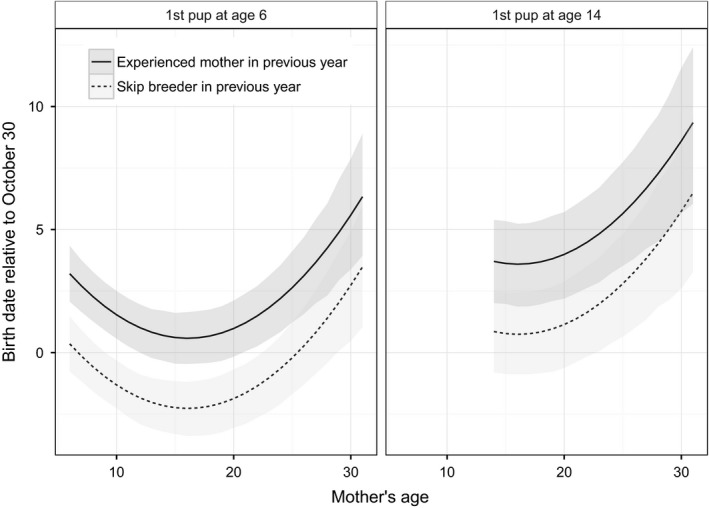
Estimates of birth dates for female Weddell seal pups born to mothers of different ages that were primiparous at either age 6 (left panel) or 14 (right panel) years of age and in either the experienced‐mother state (Exp, solid line) or the skip‐breeder state (Skip, dashed line) the previous year. Females in the experienced‐mother state in the previous year would be giving birth to at least their third pup in the current year and producing pups in back‐to‐back years. Females in the skip‐breeder state in the previous year did not produce a pup in the previous year but had reproduced at least once previously. Estimates are based on Bayesian hierarchical modeling of data from 4465 pups born from 1984 through 2014 in Erebus Bay, McMurdo Sound, Antarctica. Ages in each panel represent maternal ages in the dataset within the maternal categories presented in each panel. Gray bands present uncertainty about predictions based on the posterior distribution and display the most credible set of values that contain 95% of the posterior distribution's mass.

Age of first reproduction was positively related to birth date as we predicted (Table 1) and accounted for a difference of several days in birth dates for pups born to mothers with maximal differences in ages of primiparity (Fig 3). For example, a female pup born to a 16‐year‐old experienced mother is predicted, on average, to be born approximately 3 days earlier (95% HDI: 1.3 to 4.8) if her mother reached primiparity at age 6 vs. age 14.

A mother's reproductive state in the previous year was also related to her pup's birth date but in a simpler manner than what we had predicted. Specifically, the model results indicate that birth dates were related to whether a mother had a pup or not in the previous year but not to previous reproductive experience within those two classes (Table 1). Females that had not given birth in the previous year (Pre or Skip) typically gave birth earlier than those that had produced pups in the previous year. For example, a female pup born to a 16‐year‐old mother with an age at primiparity of 7 was predicted to be born on 27 October (26 October to 28 October) if her mother had skipped reproduction in the previous year and 30 October (29 October to 31 October) if she had produced a pup (Fig 3).

The coefficients estimated for the deterministic portion of the model had 95% HDI's that did not include zero or only slightly overlapped zero (Table 1). The typical effect sizes associated with changing a given covariate's value by 2 SD were estimated to be ~2–5 days. In total, the deterministic portion of the model (fixed effects) explained a modest portion of the total variance in birth dates (${\hat{R}}_{\mathrm{marginal}}^{2}$ = 0.055). In contrast, the total amount of variance explained by both the stochastic (random effects) and deterministic portions of the model was markedly higher (${\hat{R}}_{\mathrm{conditional}}^{2}$ = 0.60). The amount of variance associated with maternal identity (${\hat{\sigma }}_{\mathrm{mom}}^{2}$ = 25.21) was greater than the amounts associated with years (${\hat{\sigma }}_{\mathrm{year}}^{2}$=3.77) or fixed effects (${\hat{\sigma }}_{\mathrm{fix}}^{2}$ = 2.95) or the amount that remained unexplained by the model (${\hat{\sigma }}_{y}$ = 21.56).

**Figure 4:**
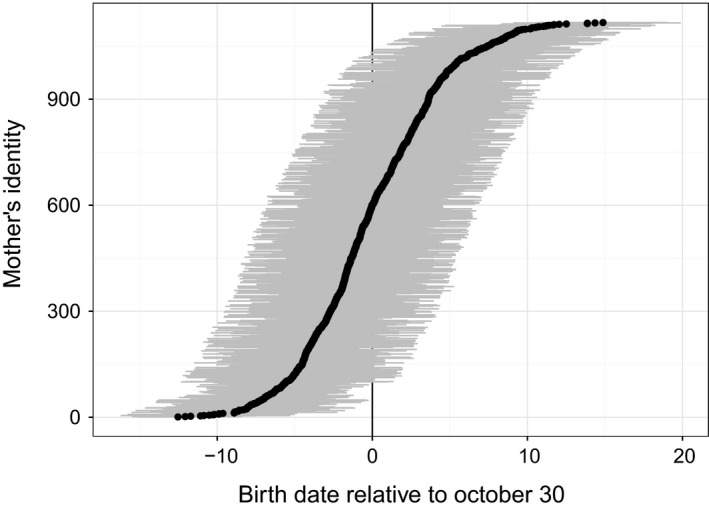
Predicted birth dates for individual Weddell seal mothers based on estimated random effects of maternal identity from a Bayesian hierarchical model of Weddell seal birth dates in Erebus Bay, McMurdo Sound, Antarctica. Estimates were based on repeated observations of birth dates for mothers in multiple years (mean number of birth dates per mother was 4, SD = 2.9, range = 1–18). Predicted birth dates for individual mothers ranged from 12.5 days before to 14.9 days after the mean date for all mothers. Gray bands present uncertainty about predictions based on the posterior distribution and display the most credible set of values that contain 95% of the posterior distribution's mass.

**Figure 5:**
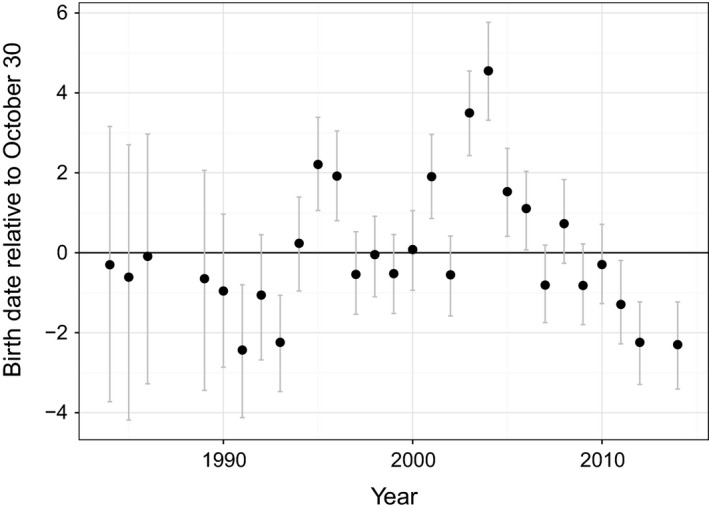
Predicted yearly departures from the overall average birth date of October 30 for Weddell seals in Erebus Bay, McMurdo Sound, Antarctica. Estimates were based on estimated random effects of year from a Bayesian hierarchical model of data from 4465 pups born in 28 different years during 1984–2014 (mean number of birth dates available per year = 159.5, SD = 102.7). In 2003 and 2004, which were the third and fourth years of a 5‐year period when four massive fragments of an iceberg were in the vicinity of the pupping colonies, the annual averages were predicted to be 3.5 and 4.6 days later, respectively, than the long‐term average. Gray bands present uncertainty about predictions based on the posterior distribution and display the most credible set of values that contain 95% of the posterior distribution's mass.

Predicted random effects for individual mothers ranged from −12.5 days (95% HDI: −15.1 to −9.9) to 14.9 days (95% HDI: 10.7–19.0), and predicted birth dates were 7 days earlier and 8 days later than the mean for 5% of mothers at either end of the distribution (Fig 4). Predicted yearly departures from the overall average birth date were ≤~2 days for all but 2 years (Fig 5). In 2003 and 2004, which were the third and fourth years of a 5‐year period when 4 massive fragments of an iceberg were in the vicinity of the pupping colonies, annual averages were predicted as being 3.5 (95% HDI: 2.4–4.6) and 4.6 days (95% HDI: 3.3–5.8) later, respectively, than the long‐term average, which were similar to empirical estimates of departures form the long‐term average for those years (3.2 [SE = 0.5] and 4.3 [SE = 0.8] days, respectively).

## Discussion

Our results shows that birth dates in the world's southernmost mammal tend to be quite synchronized but can vary by up to 49 days and depend strongly on a mother's characteristics. In agreement with numerous other studies (Reiter et al. 1981; Lunn and Boyd 1993; Boltnev and York 2001; Robbins et al. 2012; Plard et al. 2014), we found evidence that females in the best condition tend to give birth earlier in this population. Specifically, birth dates were earliest for pups born to prime‐age mothers that began producing earlier in life and that had not produced a pup in the previous year. In addition, birth dates were later during years when massive icebergs disrupted the area's sea‐ice dynamics, food web, and seal reproduction (Seibel and Dierssen 2003; Chambert et al. 2012). Although we previously found that later‐born pups have higher survival rates during the preweaning period (Proffitt et al. 2010), we suspect that postweaning survival is higher for early‐born pups because they tend to have higher‐quality mothers and can grow and gain more experience before the sea ice begins to break up in the latter portion of the pupping season and pups become vulnerable to killer whales (*Orcinus orca*, Pitman and Durban 2012). Further, because preweaning survival is high in our population (Proffitt et al. 2010), whereas postweaning survival is not (Rotella et al. 2012), we suspect that selection favoring postweaning survival is more important. Evaluating how early‐life survival varies with birth date will require future analyses, but the results reported here indicate that diverse sources of variation in birth date exist such that those analyses should be informative.

In contrast with numerous studies of large mammals that have found birth date to be negatively related to maternal age (Reiter et al. 1981; Lunn and Boyd 1993; Boltnev and York 2001; Haskell et al. 2008; Wolcott et al. 2015), we found evidence of a parabolic relationship where birth dates were latest for younger and older mothers and earliest for prime‐age mothers. Sydeman et al. (1991) reported a similar pattern for birth dates in northern elephant seals as they aged, and especially as they gained reproductive experience. (In our data, age and reproductive experience were so strongly correlated that we could not disentangle their effects.) In our study, the earliest birth dates were for pups born to mothers that were ~16 years of age, which is within 1 year of the age when reproductive rates are at their highest (Chambert et al. 2013) and just prior to the onset of physiological (Hindle et al. 2009) and body mass (Proffitt et al. 2007) senescence. We speculate that prime‐age females are more efficient at acquiring nutritional resources than younger or older females, which is in keeping with age‐related patterns of resource acquisition across numerous mammal species (Gaillard et al. 2000). This idea is also in keeping with what has been reported for age‐related changes in body mass in our study population. Specifically, age‐related improvements and subsequent senescent declines in body mass occur in Weddell seal mothers (Proffitt et al. 2007). We speculate that such changes are partly responsible for the similar, age‐related pattern we found for birth dates given that body condition can influence the timing of implantation and birth in pinnipeds (Boyd 1991). We do not, however, know how well body mass performs as a measure of body condition and did not have measures of body mass for the individual mother's studied here.

A female's reproductive state in the previous year, a covariate that we predicted would be directly related to female body condition during pregnancy, was also related to birth date. Weddell seal mothers rely heavily on stored body reserves during the 5‐ to 6‐week lactation period and lose, on average, ~40% of their postpartum mass (Boness and Bowen 1996; Wheatley et al. 2008a, 2008b). Thus, we predicted that females that reproduced in the previous year would not be able to give birth as early as other females. Our results supported the prediction and are in keeping with similar findings for red deer (*Cervus elaphus*, Guinness et al. 1978a, 1978b; Clutton‐Brock et al. 1983) and bighorn sheep (*Ovis canadensis*, Feder et al. 2008). In contrast, birth dates for Antarctic fur seals (*Arctocephalus gazella*) did not depend on a female's parturition status in the previous year (Boyd 1996), and parturition dates in roe deer (*Capreolus capreolus*) were rather insensitive to female condition (Plard et al. 2014).

We had further predicted that mothers that were primiparous in the previous year would give birth later than those that were multiparous, but our results did not provide evidence of a difference. Our prediction was based on our earlier findings that primiparous mothers incur higher costs of reproduction and are less likely to reproduce again the following year when compared to more experienced mothers (Hadley et al. 2007; Chambert et al. 2013). However, we recently found important levels of individual heterogeneity in the probability of reproduction among females (Chambert et al. 2013, 2014). Further, recent results for red deer (Stopher et al. 2008) and roe deer (Plard et al. 2014) indicate that females of higher quality tend to give birth early. Thus, we speculate that birth dates for primiparous and multiparous mothers are similar because the subset of females that achieves the challenging task of giving birth in the year following primiparity contains a higher‐than‐average proportion of high‐quality females, which tend to give birth early. Although future analyses will be required to evaluate this speculation, we did find other evidence that mothers of higher quality give birth early using two time‐invariant characteristics of mothers.

Specifically, we found support for our prediction that birth dates would be earlier for females with earlier age of first reproduction, a covariate linked to individual quality in Weddell seals (Hadley et al. 2006) and northern elephant seals (Reiter and Le Boeuf 1991). Also, birth dates were repeatable for individual mothers, which provides further evidence that female quality (as expressed by birth date) varies in the population. Female identity explained a large amount of variation in the data and predicted birth dates for individuals differed by as much as 27 days. Our findings add to a small but growing number of large‐mammal studies reporting repeatability of birth dates for individuals (Lunn and Boyd 1993; Boyd 1996; Ellis et al. 2000; Cordes and Thompson 2013; Plard et al. 2013, 2014; Wolcott et al. 2015) and lend support to recent studies that reported that fixed maternal traits account for more of the variation in birth dates than do dynamic attributes (Plard et al. 2014; Wolcott et al. 2015).

In light of the diverse evidence that we obtained indicating that females in the best condition tend to give birth early, our finding that male pups tend to be born earlier provides support for the Trivers and Willard (1973) sex‐allocation model. We did not, however, directly measure maternal body condition in the work presented here, and thus, it would be useful in the future to analyze birth date and pup sex data for mothers of known body condition. We had previously found that female Weddell seals with higher lifetime reproductive output were more likely to produce sons (Proffitt et al. 2008), and the results of the current study indicate that sex allocation might change over the course of a female's life, which is something we plan to evaluate in future analyses. Females in better condition might be more likely to produce sons because they have excess in utero glucose levels early in cell division, which can favor development of male blastocysts (Gutiérrez‐Adán et al. 2001; Cameron 2004): We plan to assess this possibility in the future using longitudinal data we recently began collecting. Evidence for the Trivers–Willard model has been mixed, and results depend on the nature of the study (Cameron 2004). Experimental work on reindeer (*Rangifer tarandus*) found that late‐conceiving females were in poorer condition and produced a preponderance of daughters (Holand et al. 2006). Studies of red deer (Clutton‐Brock et al. 1984) and mountain goats (*Oreamnos americanus*, Côté and Festa‐Bianchet 2000) also reported that maternal quality, as measured by social rank rather than birth date, was related to offspring sex ratio, but a study of bison (*Bison bison*, Green and Rothstein 1991), which used diverse measures of maternal condition, did not. Differential timing of birth for sons and daughters has previously been reported for gray seals (*Halichoerus grypus*, Coulson and Hickling 1961) and Weddell seals (Stirling 1971), but neither of those studies presented information on maternal quality.

Annual means and ranges for birth dates were remarkably consistent except during 2 years when birth dates were delayed. Those 2 years occurred during the period of time when a massive iceberg altered sea‐ice conditions and affected diverse aspects of the Ross Sea ecosystem (Arrigo et al. 2002; Arrigo and van Dijken 2003; Seibel and Dierssen 2003; Kooyman et al. 2007). In 2004, when birth dates were most delayed, pup production was the lowest it has ever been from 1963 to present and only 39% of the long‐term average (Siniff et al. 1977; Chambert et al. 2012). Even more extreme versions of annual variation were reported for South American sea lions (*Otaria flavescens*) in relation to El Niño – La Niña events (Soto et al. 2004). Later birth timing in response to challenging environmental conditions and reduced food availability has been reported in diverse species of ungulates and marine mammals (Pitcher et al. 2001), although there are notable exceptions and variation in results might be tied to where species fall along the income–capital breeder continuum (Plard et al. 2014).

We have found evidence of several sources of variation in birth dates in a high‐latitude marine predator. The greatest amount of variation was explained by considering female identity. We also found that birth dates varied with another fixed maternal trait (age at first reproduction) as well as with two dynamic traits (recent reproductive investment and age). When considered in combination across a suite of maternal traits, some individual mothers are predicted to give birth days to weeks apart. We predict that such differences in birth timing are likely to have biologically important effects for pup fates given that they typically gain ~2 kg/day during lactation (Wheatley et al. 2006) and plan to evaluate that prediction in future studies.

## Conflict of Interest

None declared.
